# Development of a Mobile App to Monitor the Effectiveness of a Hydrolyzed Cartilage Matrix Supplement on Joint Discomfort: Real-World Study

**DOI:** 10.2196/42967

**Published:** 2023-04-03

**Authors:** Christie Newman, Els Adriaens, Nicolina Virgilio, Sara Vleminckx, Sara de Pelsmaeker, Janne Prawitt, Catarina I F Silva

**Affiliations:** 1 Elénzia Ltd Redditch United Kingdom; 2 Adriaens Consulting Aalter Belgium; 3 Rousselot BV Gent Belgium

**Keywords:** digital tool, hydrolyzed cartilage matrix, hydrolyzed collagen, chondroitin sulfate, joint discomfort, real-world study, dietary supplement, mobile application

## Abstract

**Background:**

Joint discomfort is a widespread and growing problem in active adults. The rising interest in preventative nutrition has increased the demand for supplements reducing joint discomfort. Protocols assessing the effect of a nutritional intervention on health commonly involve a series of face-to-face meetings between participants and study staff that can weigh on resources, participant availabilities, and even increase dropout rates. Digital tools are increasingly added to protocols to facilitate study conduct, but fully digitally run studies are still scarce. With the increasing interest in real-world studies, the development of health apps for mobile devices to monitor study outcomes is of great importance.

**Objective:**

The purpose of this real-world study was to develop a specific mobile app, Ingredients for Life, to conduct a 100% digital study testing the effectiveness of a hydrolyzed cartilage matrix (HCM) supplement on joint discomfort in a heterogeneous group of healthy, active consumers.

**Methods:**

The Ingredients for Life mobile app using a visual analog scale was specifically developed to monitor the variation in joint pain after exercise by the study participants. A total of 201 healthy and physically active women and men (18-72 years old) with joint pain completed the study over a period of 16 weeks. Participants were randomly allocated to the study groups and did not receive any dietary or lifestyle advice. Each participant indicated one area of joint pain and logged the type and duration of their weekly activities. They received blinded study supplements and took a daily regimen of 1 g of HCM (HCM group) or 1 g of maltodextrin (placebo group) for 12 weeks while weekly logging joint pain scores in the app. This was followed by a 4-week washout period during which participants continued reporting their joint pain scores (until the end of week 16).

**Results:**

Joint pain was reduced within 3 weeks of taking a low dosage of HCM (1 g/day), regardless of gender, age group, and activity intensity when compared with the placebo group. After stopping supplementation, joint pain scores gradually increased but still remained significantly lower than those of the placebo group after 4 weeks of washout. The low dropout rate (<6% of participants, mainly in the placebo group) demonstrates that the digital study was well received by the study population.

**Conclusions:**

The digital tool allowed us to measure a heterogeneous group of active adults in a real-world setting (without any lifestyle intervention), thus promoting inclusivity and diversity. With low dropout rates, it demonstrates that mobile apps can generate qualitative, quantifiable, real-world data showcasing supplement effectiveness. The study confirmed that the oral intake of a low dose (1 g/day) of HCM led to a significant reduction of joint pain from 3 weeks after starting supplementation.

## Introduction

Joint discomfort is a common problem in the daily life of active people [1]. Its development is often associated with the intensity of sports activity, the performance level (elite vs recreational), previous injuries, or other health conditions that increase the likelihood of joint injury [2-4]. Joint discomfort is a widespread and growing problem with aging [5] and is more likely to be experienced by women than by men [6,7]. There are several factors that put women at a higher risk of developing joint pain, namely, anatomical and hormonal factors, as well as obesity, which occurs more frequently in women [8].

The combination of an aging population and the growing interest in preventative nutrition has increased the demand for supplements that can reduce joint discomfort in active adults. For instance, hydrolyzed collagen–based supplements have been shown to positively impact joint health [9,10] and have become increasingly popular over the years. The repurposing of these food-grade animal by-products to high-value health-promoting products adds to the development of a more sustainable food industry with a lower environmental impact [11,12].

The hydrolyzed cartilage matrix (HCM) containing collagen and chondroitin sulfate (CS) has shown positive effects on joint protection in a mouse model for obesity-accelerated posttraumatic osteoarthritis (OA) [13]. However, the effects of this natural matrix are yet to be tested in the general population.

The mode of action of collagen and CS is not fully elucidated, but several investigators have suggested direct joint protective properties [14,15]. Hydrolyzed collagen is a source of bioactive peptides with a positive impact on health [10,14,16], partly due to their ability to upregulate the synthesis of extracellular matrix proteins in various tissues [17,18]. A preclinical study demonstrated that hydrolyzed collagen stimulated the production of type II collagen and proteoglycans in cartilage [19]. Both matrix components change in relative amounts and structure in case of disease or damage and can subsequently alter the mechanical properties of cartilage [20]. Hydrolyzed collagen–derived peptides such as prolyl-hydroxyproline (Pro-Hyp) and proline-hydroxyproline-glycine (Pro-Hyp-Gly) circulate in the blood up to 4 hours after oral ingestion [21-23]. Pro-Hyp has been shown to exert chondroprotective effects in mice [17]. The positive effects of CS are linked to its anti-inflammatory activity [24,25], stimulation of proteoglycan and hyaluronic acid synthesis, and a reduction of catabolic chondrocyte activity [26].

The joint health benefits of hydrolyzed collagen and CS have been shown mainly in patients with OA [27-29] or in athletes [30]. However, dietary supplements to reduce or prevent joint discomfort increasingly attract consumers of any age, gender, and lifestyle. Nonetheless, extrapolating findings from OA clinical trials to this general population carries major limitations, and evidence acquired in real-world scenarios is scarce. Despite their less controlled designs, real-world studies have recently gained interest in the scientific community because of their high relevance for the general population [31,32]. When interpreted within their limitations, they can constructively fill the gap between the outcomes from controlled clinical trials and the expected effectiveness in real-world conditions [31]. In addition, new digital tools such as health applications for mobile devices are emerging to monitor study outcomes, further helping to provide information directly to health care professionals [33,34].

In this study, a real-world, digitally supported approach was chosen to investigate the potential benefits of the HCM in joint discomfort. This single-blind study was performed in healthy individuals of different ages and genders and practicing different sports with various intensity levels. A specific mobile app, Ingredients for Life, was developed to gather first-hand feedback from the study participants on the effectiveness of the HCM and to find potential response patterns of different populations. This mobile app was particularly relevant to run the study during the COVID-19 pandemic, when direct contact between researchers and participants was not possible. Participants did not receive any guidelines or restrictions regarding their diet, activities, or lifestyle to be able to focus on the effectiveness and relevance of the product in the general population in a real-world setting.

## Methods

### Materials

The naturally occurring matrix of collagen peptides and CS derived from hydrolyzed porcine cartilage was provided by Rousselot BV and is commercially available as Colartix (further described as HCM). The dosage form was hydroxypropyl methylcellulose capsules containing either 0.5 g of HCM or 0.5 g of maltodextrin as placebo. Both supplements had a matched appearance.

### Ingredients for Life Mobile App

The specifically developed mobile app Ingredients for Life, which was available free of charge in the app store for both Android and iOS operating systems, was promoted to people known to experience joint pain after exercise by selected ambassadors (physiotherapists, personal fitness trainers, sports coaches, and nutritionists). Potential study participants did not suffer from any diagnosed medical conditions but would seek nutritional supplementation to support joint health. Participant recruitment, allocation, data collection, and follow-up were performed through the mobile app, and participants were guided through the necessary study information (privacy policy, research protocol, criteria, supplement ingredients information, and supplementation instructions) and taken through the weekly reporting of joint pain via remote demonstrations. Upon consent, participants received the blinded study supplements with nutrition information and directions for use via post. At week 12, the app reminded the participants to continue reporting joint pain scores while no longer taking supplementation. Upon study completion, participants received an individual report via email. All communication between researchers and participants was done via the app to avoid direct contact during the COVID-19 pandemic.

### Study Population

Consumers seeking to reduce activity-related joint pain with a nutritional supplement were recruited through the networks of various sports and health clubs across the United Kingdom via the Ingredients for Life mobile app. Eligibility criteria were as follows: older than 18 years, no medical diagnosis, no medication, frequent physical activity, and not pregnant or breastfeeding. Physical activity included all types and levels from gardening, brisk walking to marathon training. Individuals already using any type of supplementation with vitamin D, curcumin or turmeric, other collagens, green mussel extract, *Boswellia*, glucosamine, CS, and hyaluronic or folic acid were excluded from the recruitment. The app was downloaded 277 times; 36 people did not meet the inclusion criteria and were notified through the app, 17 declined to participate, and 11 did not start the study for other (unknown) reasons. Therefore, 213 healthy individuals, aged 18-72 years, were randomized into the study, of whom 201 participants completed the study.

### Ethics Approval

#### Institutional Review Board Statement

The study was approved by the Reading University Independent Ethics Committee (130819-4). Because of the study method and participant criteria, the study did not fall under medical research and was defined as a real-world study using noninvasive measures.

#### Ethics Statement Regarding Human Subjects Research

Because the research was conducted via a mobile app, the Terms and Conditions and Privacy Policy section of the app addressed the research areas where ethics, exemptions, and approvals were to be considered. Participants had to confirm that they were older than 18 years and were not on any joint pain medication. Participants agreed to provide data via the app, and it was clearly explained how these data would be used. Only once the participant accepted these terms and conditions of the research and understood the privacy policy were they able to proceed through the app and partake in the research. By accepting these conditions, they were accepting to partake in the study.

#### Informed Consent Description

The participants were informed on how they could withdraw from the study at any point within the trial and how to contact the research team with any concerns they may have had. The email they were provided with, info@ingredientsforlife.com, reached the lead Ingredients for Life app researcher Christie Newman, who had control over the data and could remove anyone who requested to be removed at any point and provide them with any additional advice if necessary. This was covered within the Privacy Policy and Terms and Conditions section of the app.

#### Privacy and Confidentiality

All collection of data was compliant with the European Union General Data Protection Regulation (GDPR) compliance guidelines. All participants were informed in the Terms and Conditions section of where and how the data they provided would be used. For example, each participant was informed that they would receive a personalized report on their individual pain scores across the 16 weeks via their personal email once they had completed the trial. Further, they were notified that their personal information would be deleted and only their pain scores and qualitative data would be used anonymously in the pooled data to create the results for the overall study.

#### Compensation

Upon study completion, participants were offered the option of a £20 (US $24.14) shopping voucher and entry into a prize draw to win a luxury stay at a hotel and spa.

### Study Design

**Figure 1:**
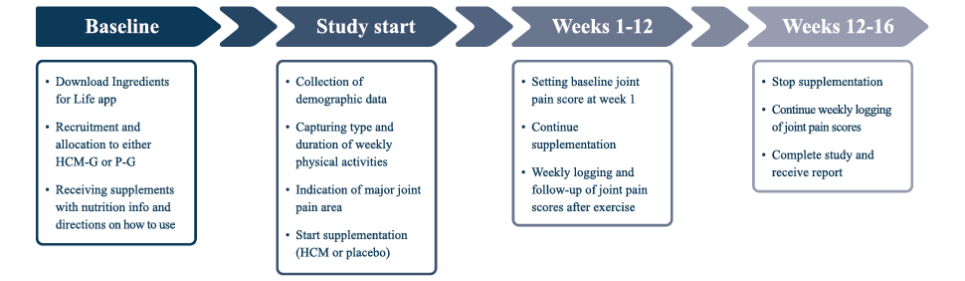
Study overview. HCM: hydrolyzed cartilage matrix; HCM-G: HCM group; P-G: placebo group.

The study was conducted in accordance with the Declaration of Helsinki. All data are protected under GDPR. The study was a 2-arm parallel design with participants randomly allocated (5:1 randomization) to either the HCM group (n=177) or the placebo group (n=36). Participants took 2 capsules with a total of 1 g of supplement or placebo per day for a duration of 12 weeks followed by a washout period of 4 weeks without supplementation but continuous logging of joint pain scores, amounting to a total study duration of 16 weeks. Participants did not receive any dietary or lifestyle advice either via a web platform or during the video calls but were given guidance on how to take the supplement and log their data. An overview of the study design is shown in Figure 1.

### Questionnaire Design

At the study start, demographic data including gender, age (in ranges), height, weight, and waist measurement were collected. The type and duration of the weekly physical activities were captured, providing a preset of choices for type (eg, gardening, brisk walking, running, cycling, tennis, swimming, strengthening sports, weightlifting [average weight squat 70 kg], flexibility or balance, yoga, Pilates, option to fill in any unlisted) and duration (eg, 0-5 hours, 6-10 hours, 11-15 hours, 16-20 hours, or >20 hours) presenting the total weekly hours of all activities combined. Each participant indicated one area of major joint pain in a body silhouette (Multimedia Appendix 1) and reported their baseline joint pain score using a visual analog scale (VAS; Multimedia Appendix 2) from 0 (pain free) to 10 (unspeakable) [35]. Each week, participants reported the level of pain using the same tool, which has been shown to retrieve accurate results on subjective pain measures of participants [36-38]. Participants’ identities were anonymized, allowing researchers to retract data at any time throughout the study if necessary.

### Weighting Matrix to Calculate the Intensity of Physical Activity

To assess the impact of physical activities of different intensity levels, a weighting matrix was developed (Multimedia Appendix 3) categorizing participants by performing low- (L), moderate- (M), and high-intensity (H) activities and by the number of performed activities (1×, 2×, 3×). Walking, swimming, flexibility or balance, gardening, cycling, dancing, and horse riding were considered activities of low joint impact. Weight lifting, gym cardio, and boxing were considered activities of moderate joint impact. Running, ball sports, skipping, and heavy weight lifting were considered activities of high joint impact. This classification was based on the UK National Health Services guidelines [39]. As an example, an LLL participant practiced 3 different types of low-intensity sports, an LLM practiced 2 different types of low- and 1 type of moderate-intensity sports, and an LLH practiced 2 different types of low- and 1 type of high-intensity sports. The other subgroups follow the same rationale. Given that only 1 participant fitted into the category MHH (1× moderate and 2× high), this participant was considered in category 5 (LHH or MMH or MHH; Multimedia Appendix 4).

### Data Analysis

The intention-to-treat population included all participants who had taken at least 1 supplement and reported at least 1 weekly VAS measure after exercise. Continuous variables were described using mean value, SD, and range, and categorical variables were described using frequencies and percentages. The effect of treatment (HCM vs placebo) on mean joint pain scores was compared with the baseline score (week 1) using a generalized linear mixed-effects (LME) model with random intercept (*b_i_*) for each participant. Follow-up time was treated as a factor variable. The full LME model with interaction is described by the following equation:

Y_ij_ = (β_0_ + b_i_) + β_1_Time_ij_ + β_2_Treatment_ij_ + β_3_Time_ij_ * Treatment_ij_ + ε_ij_

The interaction term represents the difference between the changes in the expected joint pain, comparing a participant from the HCM group with a placebo group participant for a given follow-up time. For nonsignificant interaction terms, it can be concluded that there was not enough evidence for differences in the joint pain profile over time between the supplementation groups. In that case, a second analysis was performed including only the main effects. The effect of gender on mean joint pain score was investigated by adjusting the model for gender and looking at 2-way and 3-way interaction terms between treatment, time, and gender. First, the full model was evaluated, and nonsignificant interaction terms were stepwise dropped from the model. Two exploratory subgroup analyses were performed for the HCM group only. The first analysis focused on the effect of age, and the second on the effect of physical activity intensity on mean joint pain score over time. For both analyses, a full LME model was constructed. Of note, the placebo group was not considered because the number of participants was too low for some categories of the age groups and the physical activity intensity.

Systematic departures from the normality assumptions were verified using QQ plots. Scatterplots of the residuals versus the predictions were used to check for systematic departures from the mean model. The LME analyses were performed using the *lmne* package in the R environment (R Core Team, 2021) [37]. For the main analysis, a *P* value of <.05 was considered as statistically significant. For the subgroup analyses, a *P* value of <.01 was considered statistically significant. All analyses were performed using R software (version 4.1.2; R Core Team, 2021). No imputation of missing data was performed.

## Results

### Baseline Characteristics of Study Participants

**Figure 2:**
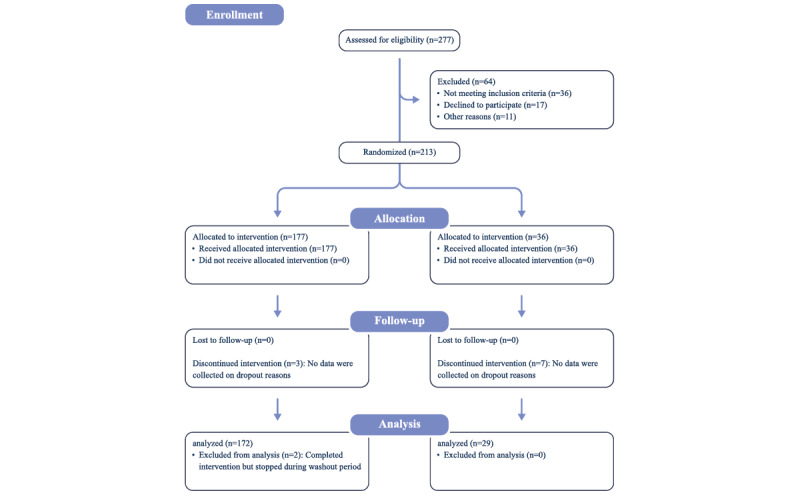
Consort flowchart.

A total of 277 individuals downloaded the app and completed the questionnaire. Of these, 36 people did not meet the inclusion criteria and were notified through the app, 17 declined to participate, and 11 did not start the study for other (unknown) reasons; 213 healthy individuals, aged 18-72 years, were randomized into the study. The consort flowchart with the study details is shown in Figure 2.

Of the 213 enrolled participants, 177 were assigned to the HCM group and 36 to the placebo group. Both groups present a similar age range (HCM group: mean 40.2, SD 11.0 years vs placebo group: mean 42.1, SD 11.0 years) and gender distribution (Table 1).

Over 90% (201/213) of enrolled participants completed the study. Notwithstanding, the percentage of participants who completed the study in the HCM group (172/177, 97%) was higher than that in the placebo group (29/36, 81%; Table 2). Although no data were collected on the reasons that led to dropout, it is important to note that of the 5 participants in the HCM group who did drop out, 2 only quit the study after week 12 and thus after the supplementation had stopped.

**Table 1:** Demographic characteristics of the study participants (N=213).

Variable		Total	HCM^a^ group	Placebo group
**Gender, n (%)**				
	Female	112 (52.6)	94 (53.1)	18 (50)
	Male	101 (47.4)	83 (46.9)	18 (50)
Age (years), (mean; range)		40.7 (11.0; 18-72)	40.2 (11.0; 18-67)	42.1 (11.0; 23-72)
**Age category (years), n (%)**				
	<30	39 (18.3)	35 (19.8)	4 (11)
	30-39	59 (27.7)	48 (27.1)	11 (31)
	40-49	69 (32.4)	57 (32.2)	12 (33)
	50-55	27 (12.7)	22 (12.4)	5 (14)
	>55	19 (8.9)	15 (8.5)	4 (11)

^a^HCM: hydrolyzed cartilage matrix.

**Table 2:** Dropout rate and group size at different time points of the study (N=213).

Study groups		Week 0, n	Week 3, n	Week 4, n	Week 5, n	Week 6, n	Week 8, n	Week 16, n	Total, n (%)
**Placebo (n=36)**									
	Completer	36	34	33	33	30	29	29	29 (81)
	Dropout	0	2	1	0	3	1	0	7 (19)
**HCM^a^ (n=177)**									
	Completer	177	177	175	174	174	174	172	172 (97)
	Dropout	0	0	2	1	0	0	2	5 (3)

^a^HCM: hydrolyzed cartilage matrix.

### Joint Pain Alleviation in the Different Supplementation Groups

**Figure 3:**
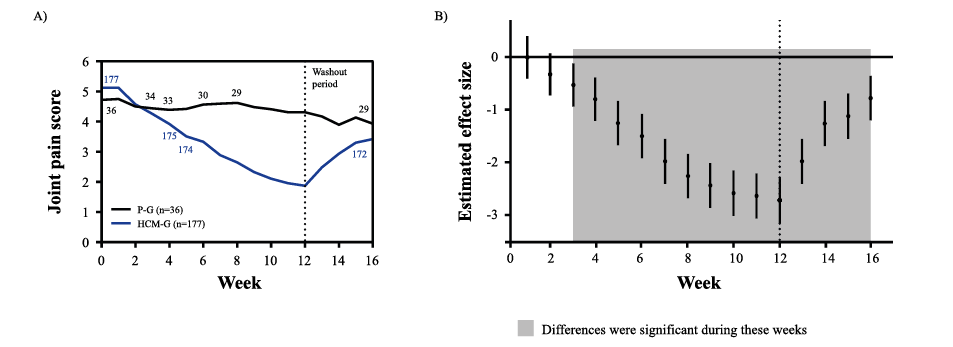
Development of joint pain scores. (A) Effect of HCM supplementation versus placebo on mean joint pain scores over a period of 16 weeks. Small numbers indicated in the graph are the number of participants in the respective study group at the given time point. (B) Estimated effect sizes and their 95% CI for the differences in mean joint pain scores between the HCM group and placebo group. Supplement intake was stopped after 12 weeks (represented by the vertical dashed line). The horizontal line represents no differences in the joint pain reported between both treatment groups. HCM-G: hydrolyzed cartilage matrix group; P-G: placebo group.

To evaluate the effect of HCM supplementation on joint pain alleviation, joint pain scores were monitored over a period of 16 weeks (Figure 3A). Statistical analysis revealed that there is a significant interaction between supplementation and time (duration on supplementation; *P*<.001), suggesting significant differences between the effect of HCM and placebo on joint pain over time (Multimedia Appendix 5).

No differences in joint pain scores were detected at baseline between both groups (*P*=.12; Figure 3B). However, this pattern changed over time. Participants in the HCM group experienced a significant reduction in mean joint pain after week 3 (*P*=.009) compared with placebo. In addition, the estimated difference in joint pain scores between the HCM group and placebo group increased over time from 0.55 (95% CI 0.13-0.96, *P*=.009, week 3) to 2.74 (95% CI 2.30-3.17, *P*<.001, week 12). Although the mean joint pain score in the placebo group remained steady over time, it decreased from 5 (distracting to moderately strong pain) to 2 (minor) in 12 weeks in the HCM group (Figure 3A).

Supplement intake was stopped after 12 weeks. Subsequently, mean joint pain scores gradually increased in the HCM group (Figure 3A). Of note, the joint pain score in the HCM group was still significantly lower than that in the placebo group at week 16 (HCM group on average 0.8 lower vs placebo group, 95% CI 0.4-1.2, *P*<.001; Figure 3B). Although the joint pain score gradually increased after stopping supplementation, the participants’ joint pain levels remained significantly below the levels reported at baseline (week 16 on average 1.7 lower vs week 0, 95% CI 1.5-1.8, *P*<.001).

### Joint Pain Alleviation in Different Genders

**Figure 4:**
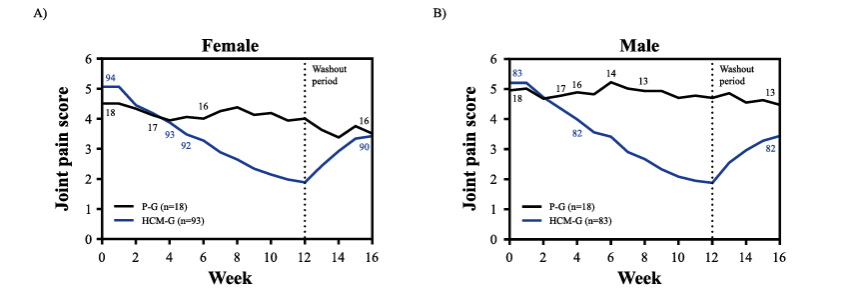
Development of joint pain scores by gender. The effect of HCM supplementation versus placebo on mean joint pain scores over a period of 16 weeks in female (A) and male (B) participants. Small numbers indicated in the graph are the number of participants in the respective study group at the given time point. Supplement intake was stopped after 12 weeks (represented by the vertical dashed line). HCM-G: hydrolyzed cartilage matrix group; P-G: placebo group.

The joint pain score development for the HCM group and placebo group by gender is depicted in Figures 4A and 4B, and dropout rates by gender are reported in Multimedia Appendix 6. The profile plots for mean joint pain scores show that there was no evidence for a significant 3-way interaction between gender, supplementation, and time (*P*>.05). No interaction between gender and supplementation nor between gender and time (*P*>.05) was recorded. Furthermore, the LME model revealed that joint pain scores were statistically similar between genders (*P*=.47).

### Joint Pain Alleviation in Different Age Groups

Several age categories in the placebo group presented only a small number of participants (Multimedia Appendix 7). Thus, the statistical analysis focused on the HCM group.

**Figure 5:**
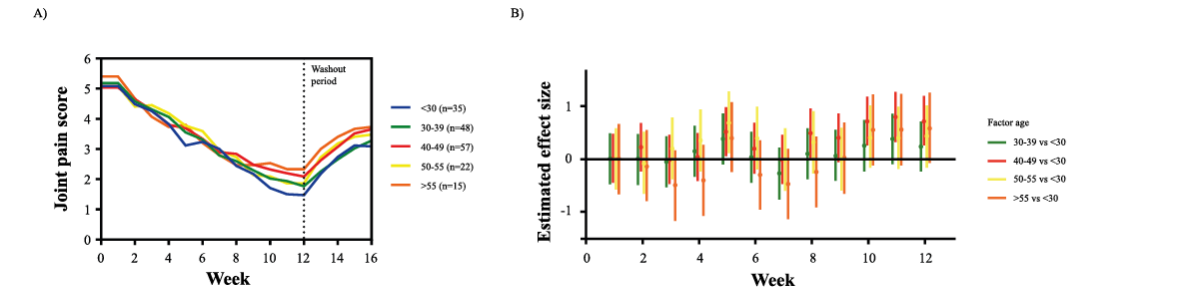
Development of joint pain scores per age category in the HCM group. (A) Effect of HCM supplementation on average joint pain scores over a period of 16 weeks per age category. Supplement intake was stopped after 12 weeks (represented by the vertical dashed line). (B) Estimated effect sizes with HCM supplementation and their 95% CI for the mean joint pain scores per age category, compared to the <30 age group. The horizontal line represents no differences in the joint pain reported between the respective age group and <30 reference group. .

The LME model revealed a significant interaction between age and time (duration on supplementation; *P*=.005), highlighting that different age categories of the HCM group differed in their joint pain levels over time (Figure 5A). Therefore, the joint pain alleviation between age categories of the HCM group was compared using the participants younger than 30 years as reference (Figure 5B). After 10 weeks on supplementation, participants aged 40-49 years had on average higher joint pain scores than did participants younger than 30 years (mean difference 0.71, 95% CI 0.25-1.18, *P*<.01; Figure 5A). This difference, however, was only recorded late in the study. Study participants younger than 30 years reported the lowest joint pain scores among all age categories. Overall, all subgroups had joint pain levels significantly below the levels reported at the baseline of the study in all age categories (Multimedia Appendix 8).

### Impact of Physical Activity Intensity on Joint Pain Alleviation

Participants were divided into 5 subgroups based on the average weekly physical activity intensity (subgroups 5 and 6 were merged because group 6 only contained 1 participant). Because of a low number of participants (Multimedia Appendix 9) and some dropouts (Multimedia Appendix 10) in the placebo group, the statistical analysis focused on the HCM group. The demographic characteristics of HCM group participants were similar across physical activity intensity subgroups (Multimedia Appendix 11).

**Figure 6:**
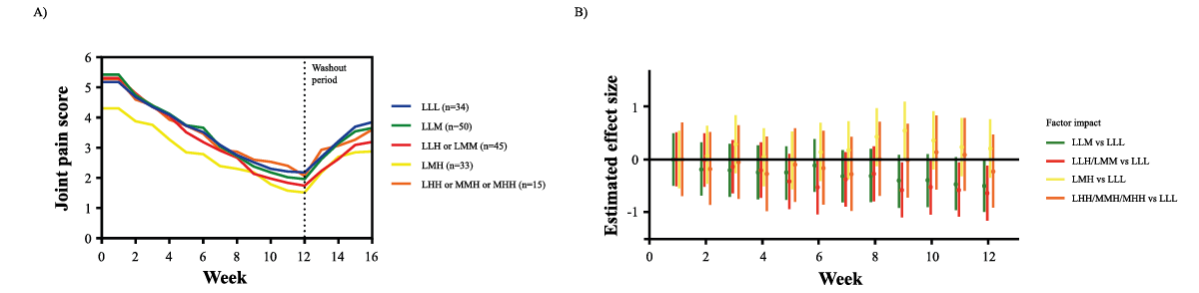
Development of joint pain scores in the HCM group per subgroup of different physical activity intensities. (A) Effect of HCM supplementation on average joint pain scores over a period of 16 weeks per subgroup of different physical activity intensities. Supplement intake was stopped after 12 weeks (represented by the vertical dashed line). (B) Estimated effect sizes and their 95% CI for each physical activity intensity subgroup compared with the LLL subgroup. The horizontal line represents no differences in the joint pain reported between the respective subgroup and the LLL subgroup. HCM-G: hydrolyzed cartilage matrix group; LHH: low-high-high; LLH: low-low-high; LLL: low-low-low; LLM: low-low-moderate; LMH: low-moderate-high; LMM: low-moderate-moderate; MHH: moderate-high-high; MMH: moderate-moderate-high.

The mean joint pain score profiles for each physical activity intensity subgroup of the HCM group are shown in Figure 6A. There is not enough evidence to conclude on a significant interaction between the physical activity intensity and time (duration on supplementation; *P*=.04). The statistical analysis revealed that joint pain scores did not vary significantly among the different subgroups of physical activity intensity (*P*=.09), suggesting that the effect of HCM supplementation is consistent across different subgroups of physical activity intensity (Figure 6B; Multimedia Appendix 11).

## Discussion

### Principal Findings

This real-world study investigated the effect of an HCM-containing hydrolyzed collagen and CS on joint discomfort in a heterogeneous group of healthy and physically active consumers in their everyday life. The study was conducted during the COVID-19 pandemic, which represented an additional challenge. The specifically designed mobile app Ingredients for Life assured the successful recruitment and follow-up of the study participants and helped to minimize the impact of the challenges caused by the pandemic. Additionally, a fully digitally run study could positively impact the diversity and inclusivity of the research by reaching groups of participants that are typically underrepresented in clinical trials. People who have mobility or transportation issues, have busy work schedules, or are single parents (often woman) have a hard time participating in face-to-face studies but can easily enter a digitally run study. The use of a mobile app to monitor the candidates’ progress overcomes the known barriers of face-to-face studies including inability to access the research center and practical and logistical issues including hidden costs (transportation, childcare, etc) [40]. This contributes to the importance of including digital tools in research.

The digital approach of this study not only allowed researchers to gather first-hand feedback from consumers on the effectiveness of the supplement but also demonstrated that mobile apps can generate qualitative, quantifiable data. Participants weekly logged their joint pain scores in the app using a VAS enabling quantification of subjective feedback. Previous studies showed that VAS is a validated pain rating scale and no clinically relevant differences exist between digitally or traditionally obtained results [41], highlighting the value and feasibility of using digital tools in scientific research. The digital execution of the study and engaging character of the app might also explain the relatively low dropout rate with over 90% of participants completing the full study.

The fact that participants were not restricted to any diet or lifestyle highlights the relevance of HCM supplementation in a real-world setting where outside factors are not controlled (contrary to strictly controlled clinical trial designs). This design allowed us to investigate if the benefits of HCM observed in an OA mouse model extend to a human heterogeneous population [13].

The results demonstrate a significant improvement of joint pain in the HCM group compared with the placebo group as early as 3 weeks after starting supplementation. Joint pain reduction further continued over the full supplementation period of 12 weeks. With the start of the 4-week washout period, where HCM supplementation was stopped, joint pain scores gradually increased but remained significantly below those of the placebo group at week 16. These findings show that daily oral HCM supplementation has a clear potential to alleviate joint discomfort in consumers of different ages and genders and performing physical activities of different intensity levels. The perceived positive effects of HCM supplementation might also explain the fact that the dropout rate in the HCM group was lower than that in the placebo group. Although dropouts are a common issue in controlled trials [42-44], the dropout rates in this study were relatively low (3% for the HCM group, 19% for the placebo group), especially given the fact that this study was run at distance.

The first preclinical report on HCM supplementation in obese mice with posttraumatic OA demonstrated cartilage protection and a significant suppression of key proinflammatory microbial strains in the gut, suggesting that HCM might indirectly protect cartilage by modulating the gut microbiome [13]. Future randomized controlled trial (RCT) protocols are warranted to assess extended clinical outcomes, capturing biomechanical function, biomarkers of inflammation, or imaging techniques to get more insights into the mode of action of HCM.

Mohammed and He [45] reported a reduction of wrist joint discomfort by a daily supplementation of 2.5 g of hydrolyzed chicken collagen type II in healthy participants aged 40-65 years (65.5% women) after 4 weeks of supplementation. Despite the differences with this study, such as RCT versus real-world approach and duration, both studies showed the effectiveness of a low dose (2.5 g vs 1 g/day).

Different factors drive the risk for developing joint pain, namely gender, age, and sports intensity [5,7,46]. Compared with previous reports that commonly investigate rather homogeneous cohorts, this study recruited participants of different ages, genders, and performing sports activities of different intensity levels. Many trials focus on groups aged >40 years, as many parameters of aging contribute to joint pain [5]. Relevant factors involve a reduction in lubricating fluids, accelerated cartilage breakdown, stiffening of ligaments, and a decrease in strength of the surrounding muscle [47]. In line with previously mentioned trials, in this study, the subgroup of participants aged 40-49 years reported a higher average joint pain score than those aged <30 years. However, all age subgroups reported a clear improvement of joint pain in response to HCM supplementation. Interestingly, although women tend to have more risk factors that determine susceptibility for joint pain, the gender had no impact on the effectiveness of HCM supplementation. HCM further showed a consistent effect across subgroups of different physical activities.

A strength of this study is the joint pain score recording during the 4-week washout period. The effect of the washout was directly noticeable in the HCM group with a gradual increase of the joint pain score over the 4-week period, while remaining constant in the placebo group, suggesting a direct mechanistic correlation between supplementation and benefit, such as an anti-inflammatory effect. CS has been reported to have anti-inflammatory activity [24,25], and so has HCM in a preclinical study [13], which could explain the attenuation of the experienced benefit in the washout period. However, joint discomfort was still lower than in the placebo group at the end of the washout period, suggesting that other, more long-lasting mechanisms, might contribute to the pain-alleviating effect of HCM. Of note, the pain scores were still decreasing at 12 weeks of HCM supplementation when the washout phase had started. Future studies should investigate whether a longer supplementation time can stabilize the pain scores at lower levels. The supplementation of placebo during a washout period might further correct for the participant’s knowledge that the supplementation has stopped and its potential influence on the joint pain scores.

Real-world studies (RWSs) provide many advantages, but, like any study design, they can also offer limitations and have to be cautiously interpreted. Compared with RCTs, patient selection and follow-up in RWS are less strict, making it harder to check patient compliance (eg, correct use of the supplement) and adding the risk of selection bias [32,48]. With the use of the Ingredients for Life app for patient selection, randomization, and follow-up, we tried to overcome these limitations. Other biases inherent to RWS, like a lack of randomization or a control supplement, were avoided by designing the study plan as a randomized, single-blind, placebo-controlled approach [32,49]. Although one of the main strengths of RWS is the large size and heterogeneity of the study population, it can also form the base of a major limitation. Diversity in participants increases data variation and might dilute the treatment effect [50], making it harder to find differences between groups; this is also called the β-error problem [49]. Test population heterogeneity can also lead to missing data [48]. This could be observed in this study, especially when dividing the data set into subgroups; the placebo groups of several age categories or activity intensities were too small to draw a trustworthy conclusion based on statistical data.

It is clear that each study design has its own strengths, and RCT and RWS could be seen as complementary, allowing the measurement of both the efficacy and effectiveness, respectively. It is important to combine data from both study types to overcome the limitations of each of them.

In general, this study shows that the digital execution of a real-world study is a promising and relevant design to assess the effects of dietary supplementation on health.

### Conclusions

To the best of our knowledge, this is the first digitally supported, real-world study showing the benefits of an HCM of collagen and CS in joint health in the general public across all age groups (18 years or older) and genders with a physically active lifestyle. This study provides evidence of the scientific value of user-friendly digital tools such as the Ingredients for Life mobile app that encourage consumer cooperation (as shown by the low dropout rate) and simplify participant tracking.

This real-world study also shows that adults of different backgrounds and lifestyles with joint discomfort, who were otherwise healthy, physically active, and not consuming other mobility-related supplementation, significantly benefited from HCM supplementation to reduce joint discomfort, regardless of age, gender, and physical activity intensity. This type of study design allows for a closer connection with the final consumer and assessing supplement effectiveness in circumstances of everyday life.

## Appendix

Multimedia Appendix 1 Silhouette of a body diagram.

Multimedia Appendix 2 Visual Analog Scale (VAS).

Multimedia Appendix 3 Weighting matrix used to categorize participants according to the type (low, moderate and high) and intensity (1x, 2x, 3x) of physical activity.

Multimedia Appendix 4 Distribution of participants according to average impact of physical activity in HCM-G and P-G. HCM-G: hydrolyzed cartilage matrix group; P-G: placebo group.

Multimedia Appendix 5 Differences in joint pain alleviation between the HCM-G and P-G. HCM-G: hydrolyzed cartilage matrix group; P-G: placebo group.

Multimedia Appendix 6 Participants’ dropout rate along the trial by gender.

Multimedia Appendix 7 Participants’ dropout rate along the trial by age category.

Multimedia Appendix 8 Profiles of average joint pain score for HCM and placebo groups by age category. HCM: hydrolyzed cartilage matrix.

Multimedia Appendix 9 Demographic characteristics of study participants of HCM-G and its distribution across subgroups of physical activity. HCM-G: hydrolyzed cartilage matrix group.

Multimedia Appendix 10 Participants’ dropout rate along the trial by physical activity.

Multimedia Appendix 11 Profiles of average joint pain score for HCM and placebo groups by physical activity impact. HCM: hydrolyzed cartilage matrix.

## Abbreviations

CS: chondroitin sulfate
GDPR: General Data Protection Regulation
HCM: hydrolyzed cartilage matrix
LME: linear mixed-effects
OA: osteoarthritis
Pro-Hyp: prolyl-hydroxyproline
Pro-Hyp-Gly: proline-hydroxyproline-glycine
RCT: randomized controlled trial
RWS: real-world study
VAS: visual analog scale

## Data Availability

The data are not publicly available due to General Data Protection Regulation (GDPR) restrictions and privacy policies outlined in the participant recruitment. The data sets generated or analyzed during this study are available from the corresponding author on reasonable request.
