# Supplementary figures and images for: Development of a Mobile App to Monitor the Effectiveness of a Hydrolyzed Cartilage Matrix Supplement on Joint Discomfort: Real-World Study
Source: JMIR Form Res. 2023 Apr 3;7:e42967. doi: 10.2196/42967 (PMC10131938; doi:10.2196/42967)

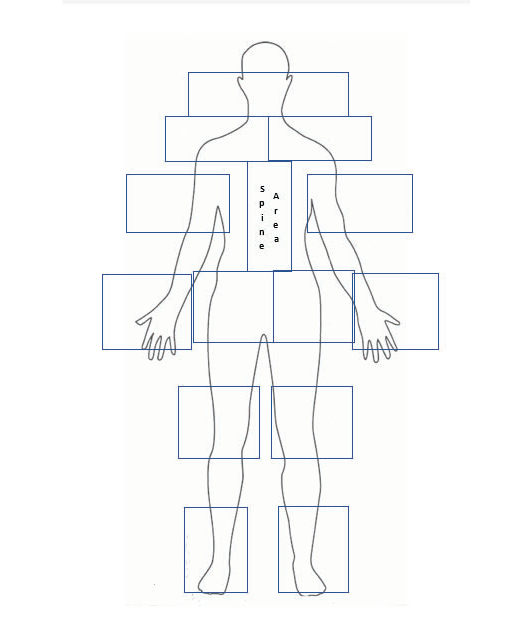

Supplement: Multimedia Appendix 1 [file formative_v7i1e42967_app1.png]

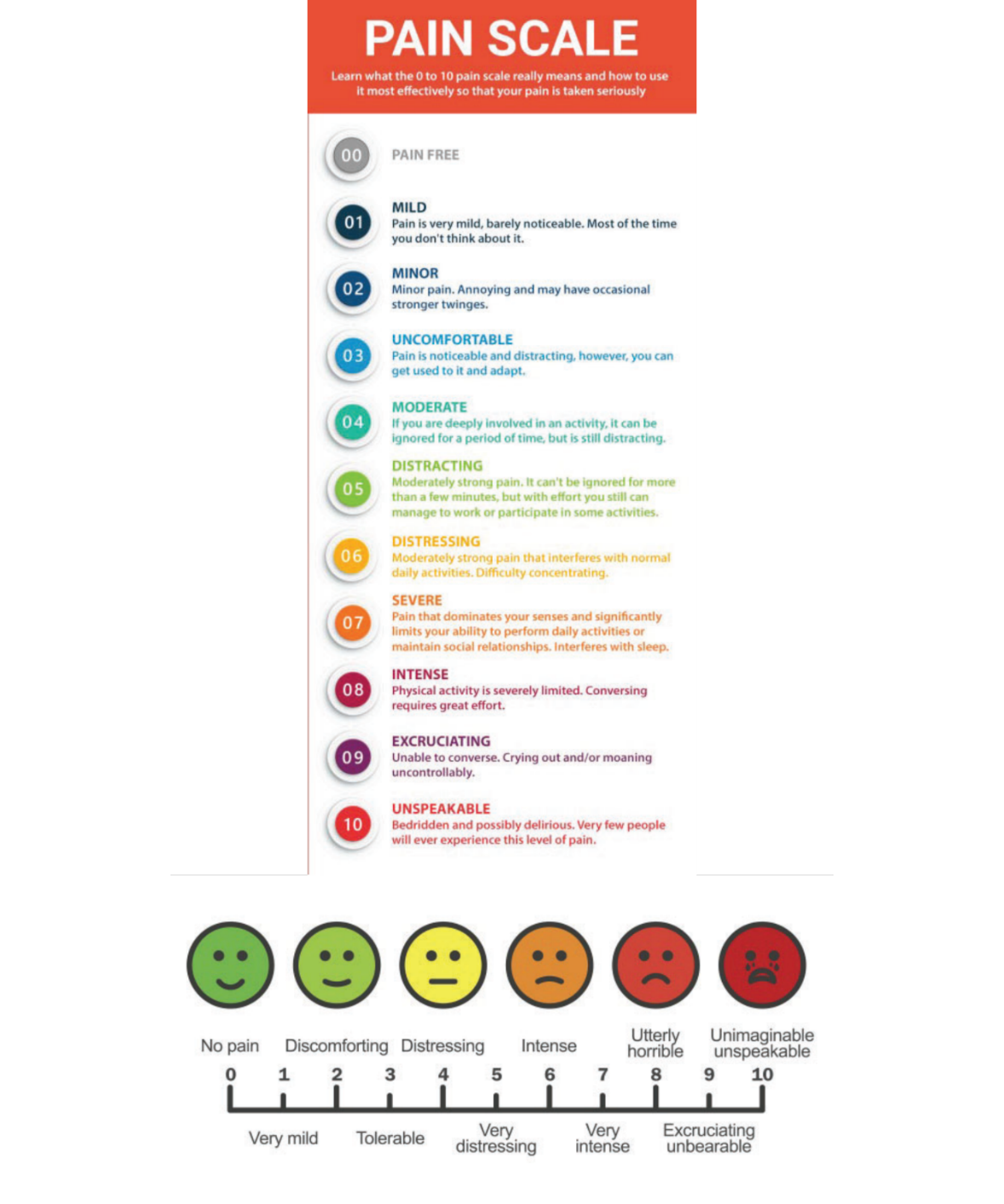

Supplement: Multimedia Appendix 2 [file formative_v7i1e42967_app2.png]

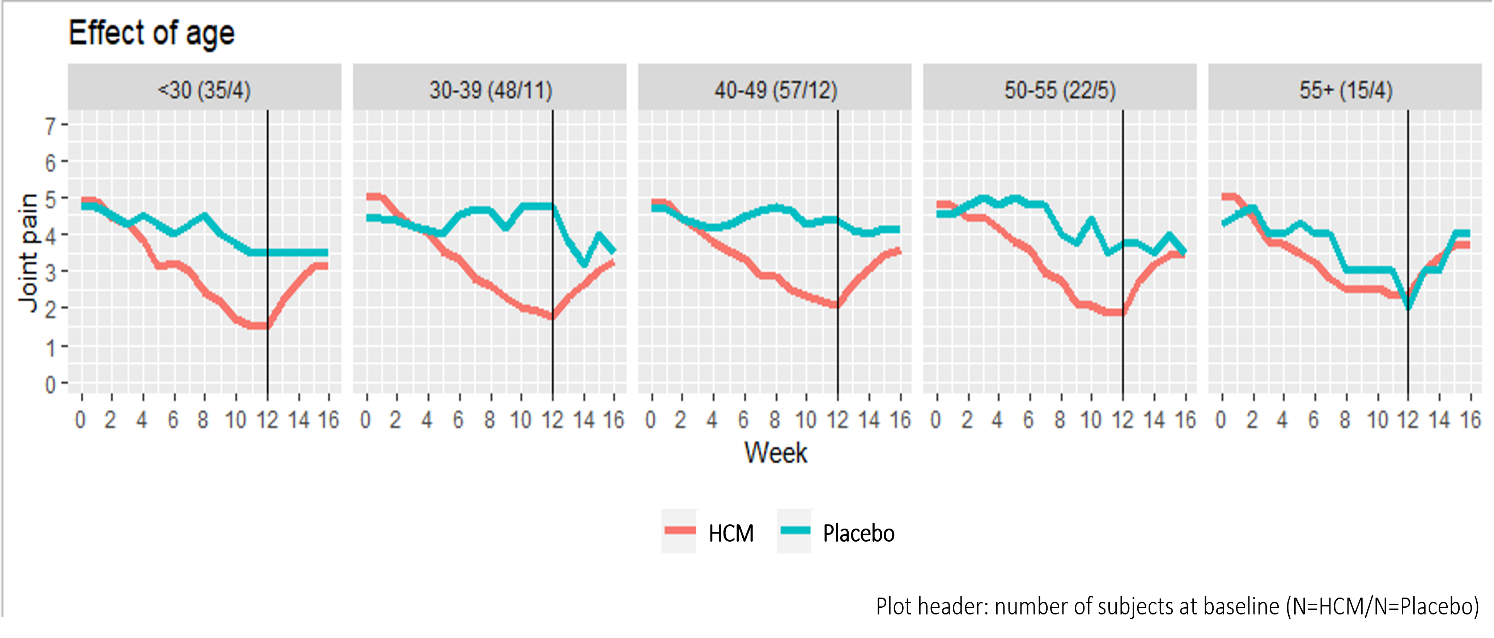

Supplement: Multimedia Appendix 8 [file formative_v7i1e42967_app8.png]

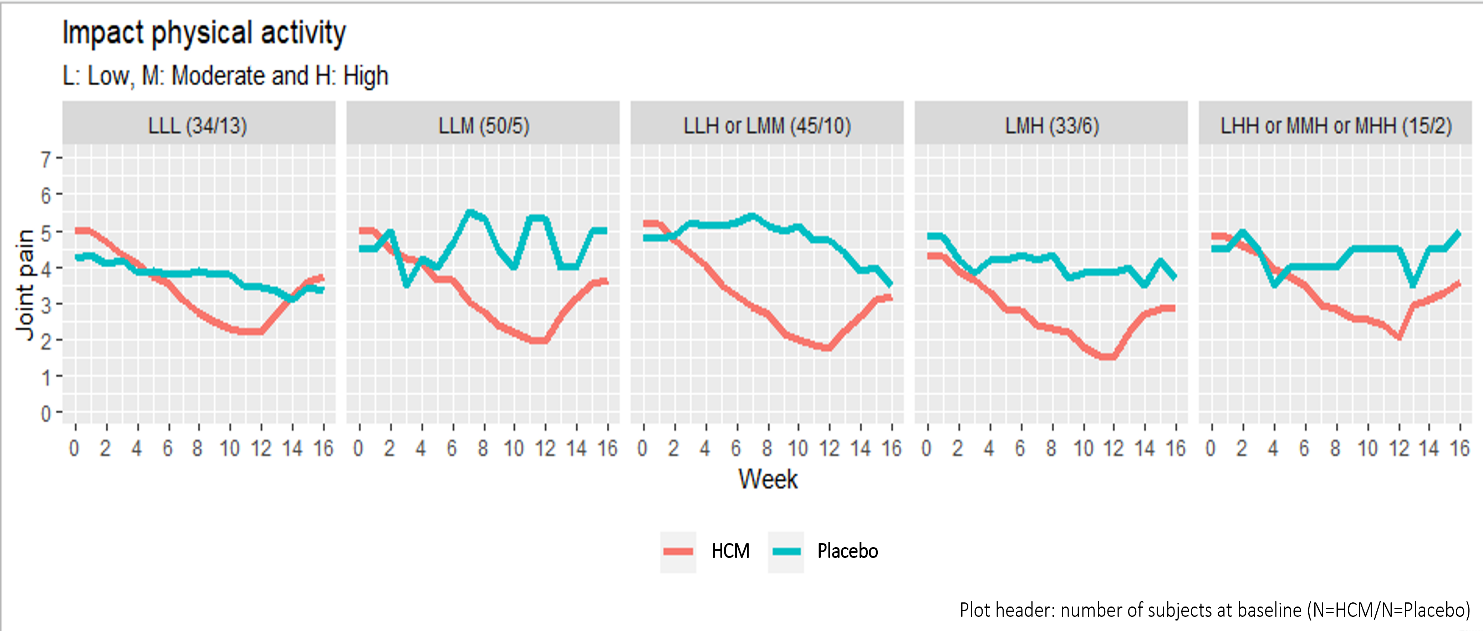

Supplement: Multimedia Appendix 11 [file formative_v7i1e42967_app11.png]
